# Supplementary material for: Quinacrine-Induced Autophagy in Ovarian Cancer Triggers Cathepsin-L Mediated Lysosomal/Mitochondrial Membrane Permeabilization and Cell Death
Source: Cancers (Basel). 2021 Apr 21;13(9):2004. doi: 10.3390/cancers13092004 (PMC8122252; doi:10.3390/cancers13092004)
Supplement: Supplementary file 1 [file cancers-13-02004-s001.zip › cancers-1157217-supplementary.pdf]

cancers MDPI

Supplementary Materials

Quinacrine-induced autophagy in ovarian cancer triggers Cathepsin-L mediated lysosomal/mitochondrial membrane permeabilization and cell death

Prabhu Thirusangu, **Christopher L. Pathoulas, Upasana Ray, Yinan Xiao, Julie Staub, Ling Jin, Ashwani Khurana and Viji Shridhar***

Table S1: Source of Materials Used

Sl no	Name of the Chemical/Reagent/Plasmid/Antibody	Catalog Number/ RRID	Source
1	Quinacrine (QC) or quinacrine dihydrochloride	Q3251	Sigma-Aldrich
2	3-methyladenine	M9281	
3	CA-074Me	#18469	Cayman Chemical
4	Pepstatin A	sc-45036	Santa Cruz Biotechnology Inc.
5	Z-FY(tBU)-DMK	sc-222423	
6	Anti-p62	sc-28359/ RRID:AB_628279	
7	anti -CTSL	sc-32320/ sc-6498 RRID:AB_626811/ RRID:AB_2245603	
8	anti-CTSB	sc-365558/ RRID:AB_10842446	
9	anti-CTSD	sc-374381/ RRID:AB_10991327	
10	anti-Bcl-w	sc-6418/ RRID:AB_2290499	
11	anti- anti-PCNA	sc-56/ RRID:AB_628110	
12	anti-β actin	sc-517582/ RRID:AB_2833259	
13	anti- α tubulin	sc-5286/ RRID:AB_628411	
14	Z-VAD-FMK	#60332	Cell Signaling Technology
15	Bafilomycin	#54645	
16	Anti-LC3B	#3868/RRID:AB_2137707	
17	anti- PARP	#9541S/ RRID:AB_331426	
18	anti-cyt c	#11940/ RRID:AB_2637071	
19	anti- cleaved capsase-9	#9509/ RRID:AB_2073476	
20	anti- cleaved caspase-3	#9664/ RRID:AB_2070042	
21	anti-mcl1	#4572S/RRID:AB_2281980	
22	and anti-Bim	#2819/ RRID:AB_10692515	
23	anti-bad	#9292 / RRID:AB_331419	
24	cell lysis buffer	#9803S.	
25	Anti-Bid	#50351-T60	Sino Biological
26	anti-myc tag	05-419	Millipore-Sigma
27	shCTSL -TRC1.5 plasmid	SHCLNG-NM_001912	Sigma Aldrich
28	pcDNA3.1-hCathepsin L construct	#11250/ RRID:Addgene_11250	Addgene
29	recombinant human CTSL (rCTSL) protein	952-CY	R&D Systems
30	recombinant human CTSB (rCTSB) protein	10483-H08H	Sino Biological
31	MitoTracker® Red FM	M22425	Life Technologies
32	LysoTracker®	L12492	ThermoFisher
34	Magic Red® Cathepsin L Assay	#942	ImmunoChemistry Technologies LLC

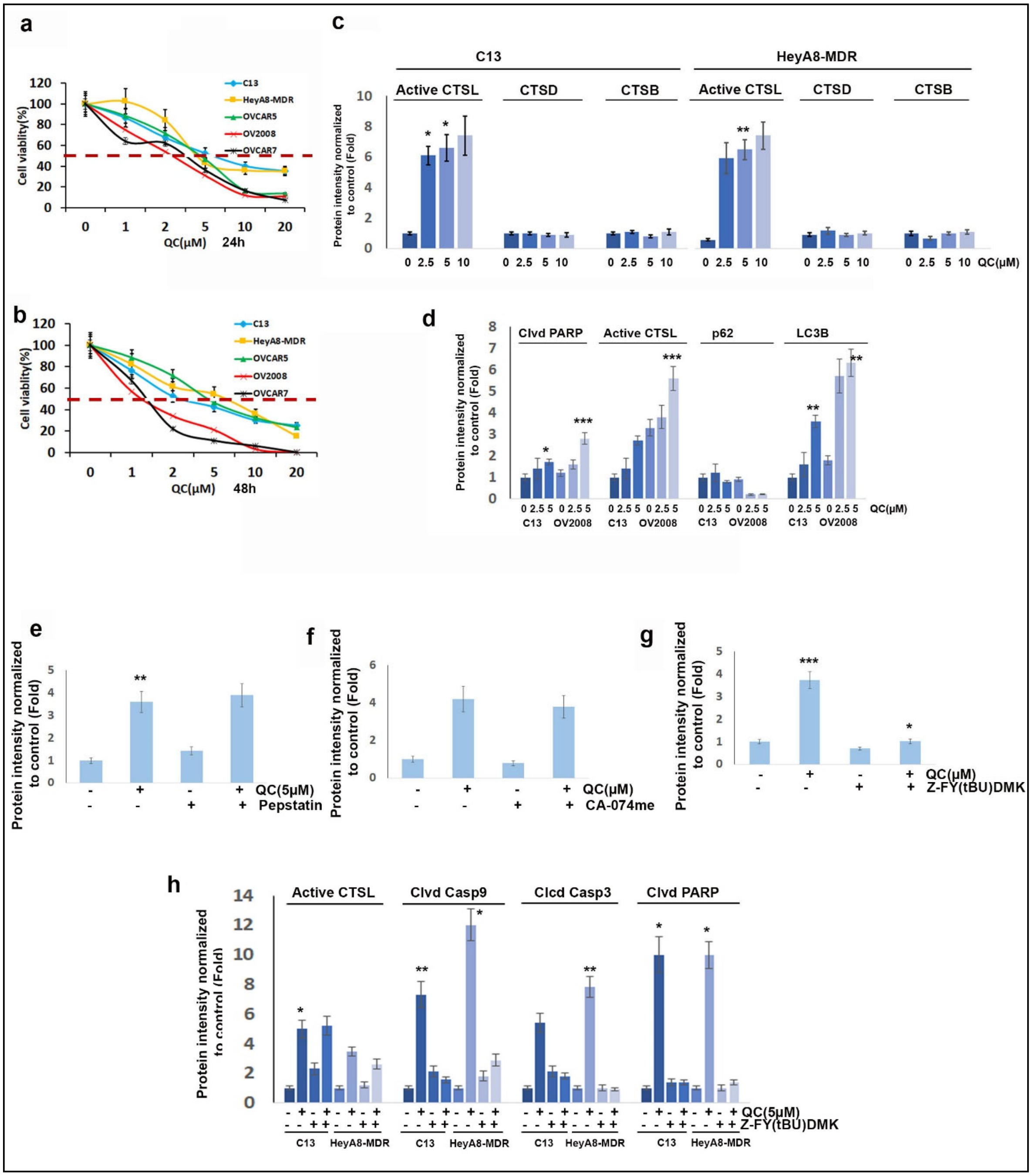

Figure S1: QC induces potential cytotoxicity against OC cells**. (a** and **b)** Cytotoxicity was determined by MTT assay on different OC cells at 24h and 48h respectively. **(c** and **d)** Graphs show the densitometric analysis for fig.1**e** and **g** respectively. **(e–h)** Densitometric plots for figures.2**e**,**f**,**i** and **j** respectively. (Statistical significance was analyzed by comparing test sample with untreated of same group and significance was expressed as $*p < 0.05$ $**p < 0.01$ $***p < 0.001$). Abbreviations: QC- Quinacrine CTSL- Cathepsin L, CTSB- Cathepsin B, CTSD- Cathepsin D, LC3B- Light Chain 3B

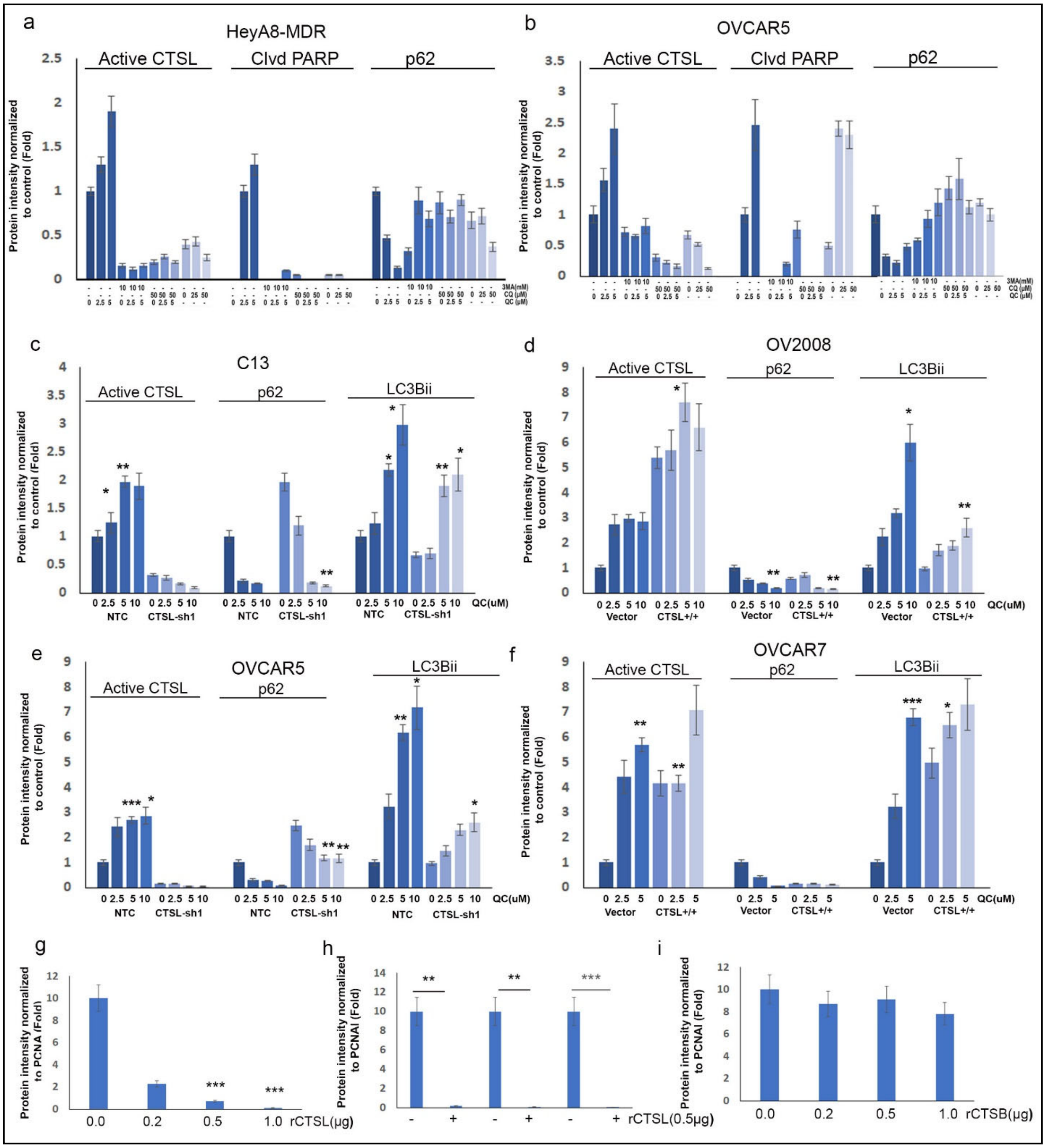

Figure S2. Densitometric analysis of protein expression normalized to control protein. **(a** and **b)** Densitometric graphs for figures 3**c** and **d**. **(c–f)** Graphs show normalized protein expression to control for figures.5a-d respectively**. (g–i)** Histographs show densitometric values for figures.5**g–i**. (Statistical significance was analyzed by comparing test sample with untreated of same group and significance was expressed as *$p < 0.05$ **$p < 0.01$ ***$p < 0.001$) QC-quinacrine, CTSL- cathepsin-L, p62- ubiquitinbinding protein 62, Cyt c- Cytochrome-c, ATG5- Autophagy Related 5, LC3B- Autophagy marker protein Light chain 3B,

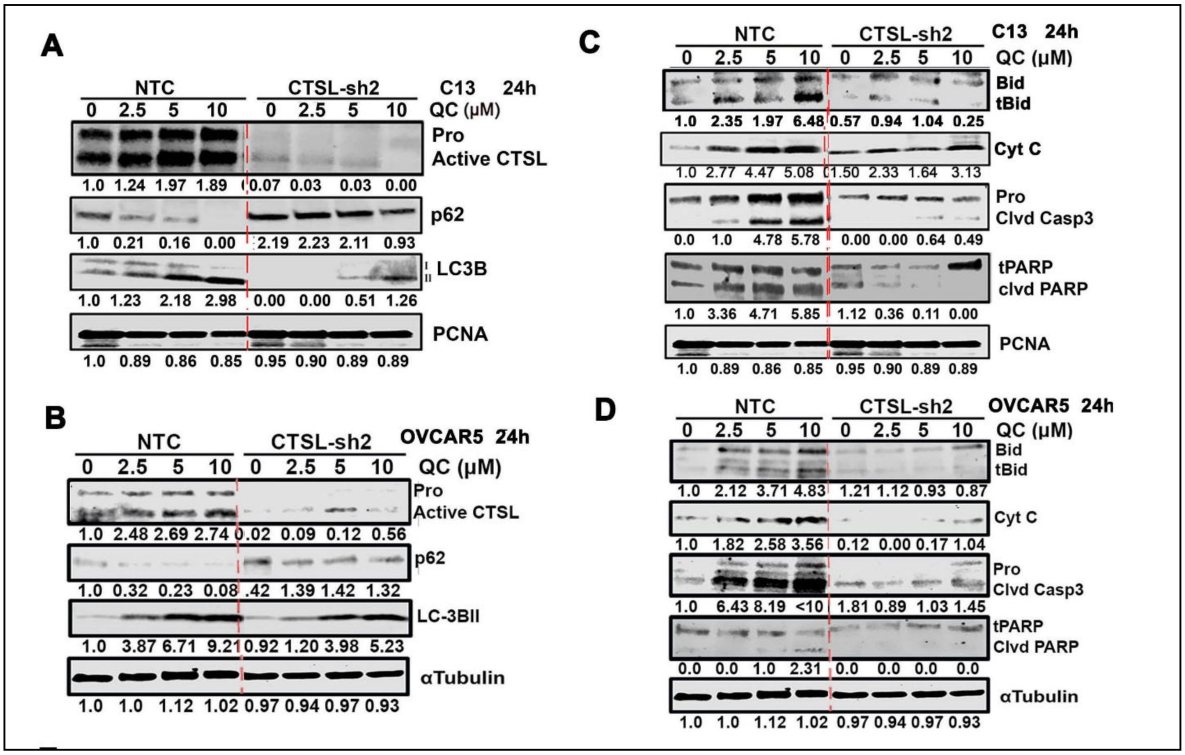

Figure S3: QC upregulated CTSL drives the autophagic flux by degrading p62 and cell death. (**a** and **b**) Western analysis in the NTC and CTSL KD sh2 clone of C13 and OVCAR5 cells upon treatment with 2.5, 5 and 10µM QC for 24hrs against CTSL, LC3BII and p62 proteins. (**c** and **d**) Similar immunoblot analysis in in the NTC and CTSL KD sh2 clone of C13 and OVCAR5 cells upon treatment with 2.5, 5 and 10µM QC for 24hrs against tBid, Cyt-C, cleaved caspase3 and PARP proteins. Abbreviations: QC-quinacrine, CTSL- cathepsin-L, tBid- truncated Bcl-2 family member(Bid), p62- ubiquitinbinding protein 62, Cyt c- Cytochrome-c, ATG5- Autophagy Related 5, LC3B- Autophagy marker protein Light chain 3B, PARP- Poly (ADP-ribose) polymerase.

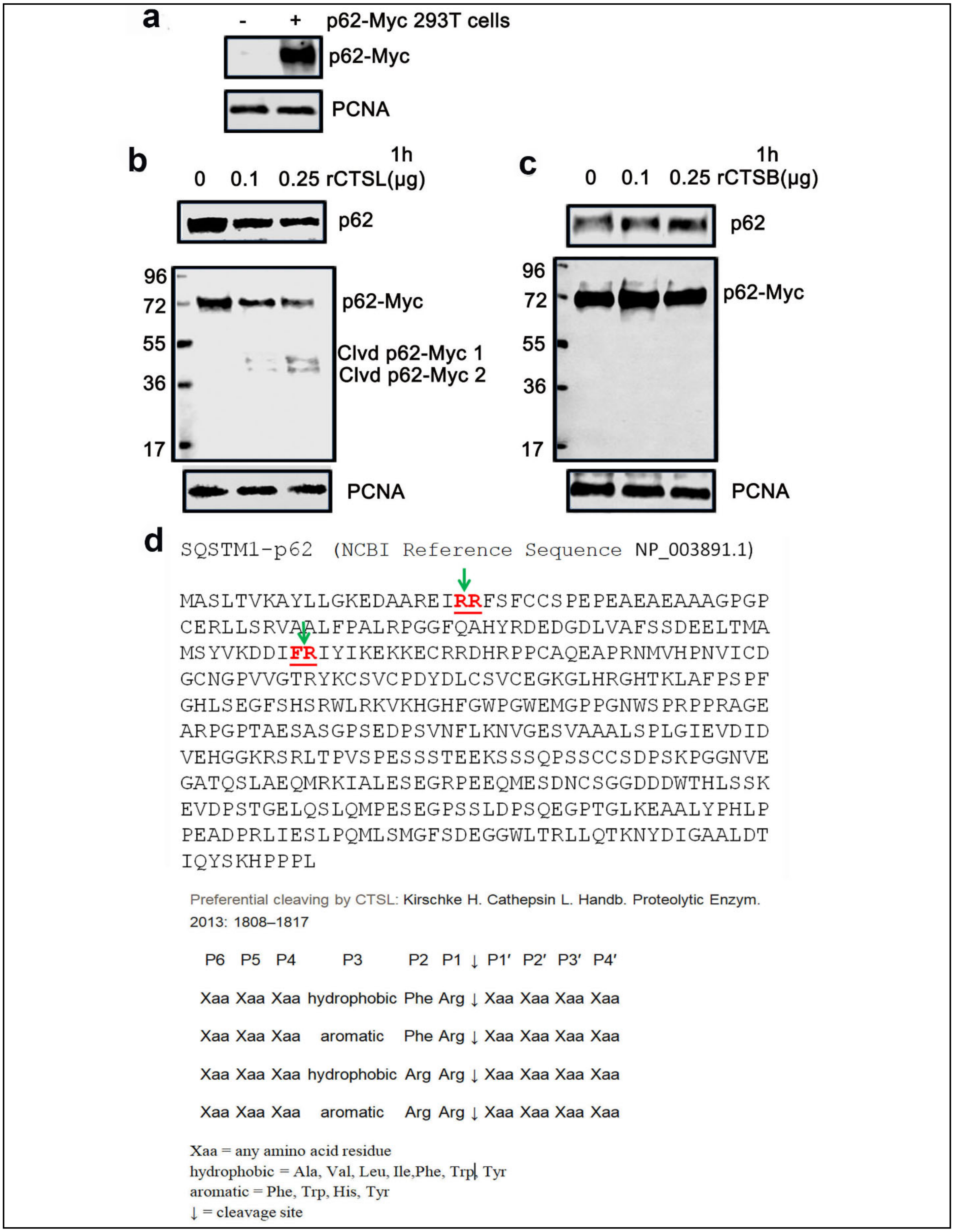

Figure S4: Recombinant CTSL cleaves autophagic key cargo protein p62 in Hek293T cells. (**a**) Myc tagged p62 protein was ectopically expressed in Hek293T cells. Western blot was performed by incubating ectopically expressed myc-p62 with rCTSL and/or rCTSB at 0.0, 0.1, 0.25μg/sample for 1h and (**b**) rCTSL cleaves ectopically expressed myc tagged p62 at two sites whereas (**c**) rCTSB incubation exhibits intact myc-p62 protein. PCNA was used as a loading control in all the westerns. (**d**) Identification of site of possible peptide cleavage of p62(SQSTM1) by CTSL cleaving site demonstrated by Kirschke,2013 [34].

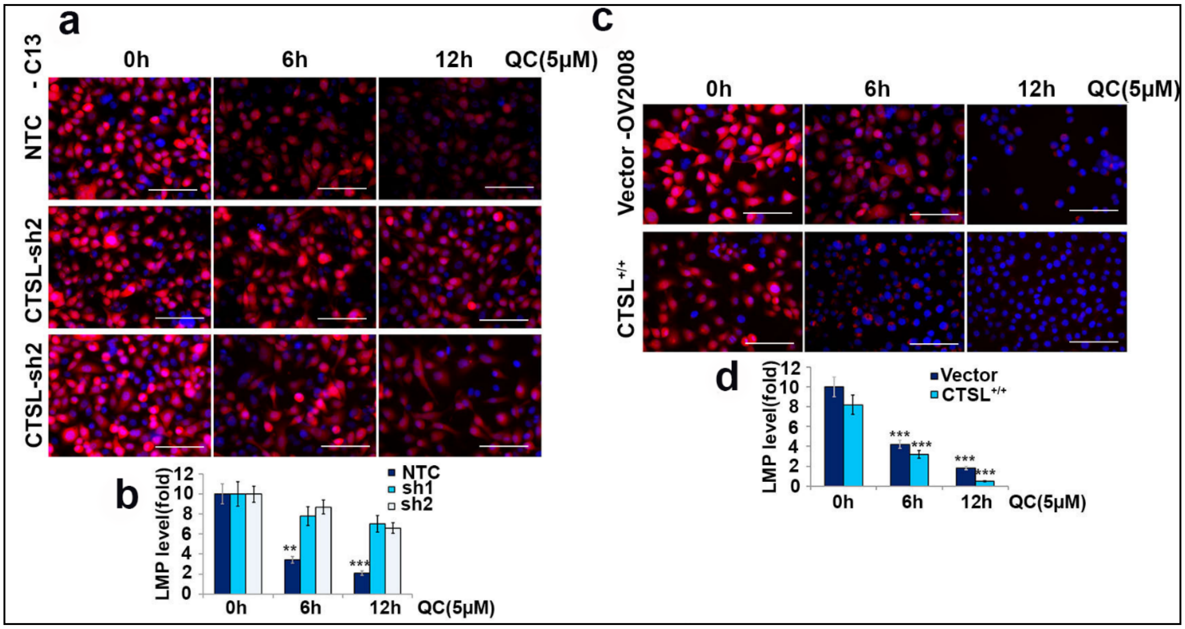

Figure S5: QC induces CTSL mediated LMP. Representative images of LMP induction in (**a**) CTSL KD sh1/sh2 and NTC control transfected C13 cells were exposed to 5μM QC for 6 and 12hrs and labelled with lysotracker Red and Hoechst33342 (blue) for nuclei.(**b**) quantitation of loss of red signal /indicative of LMP. (**c**) Similar study was executed in OV2008 control vector and CTSL-overexpressed cells and representative images provided. (**d**) Graphical representation was provided. (Scale bar – 200 μm and 40× magnifications and Statistical significance was analyzed by comparing test sample with untreated of same group and significance was expressed as *$p < 0.05$ **$p < 0.01$ ***$p < 0.001$).

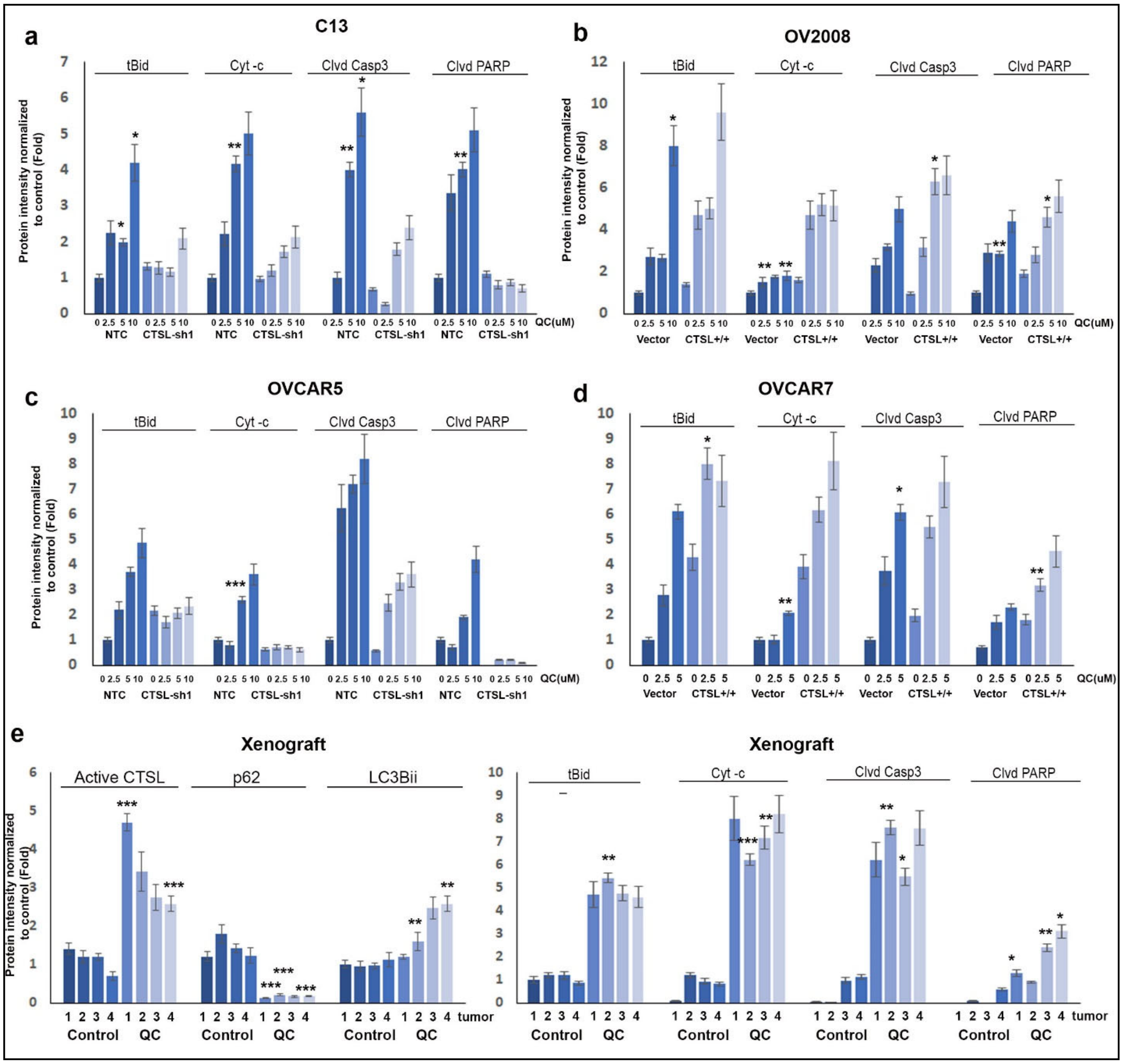

Figure S6. Densitometric analysis of protein expression normalized to control protein. **(a–d)** Densitometric graphs for figures 6g-j respectively. **(e)** Graphs show normalized protein expression to control for figure 7a of xenograft (Statistical significance was analyzed by comparing test sample with untreated of same group and significance was expressed as *p < 0.05 **p < 0.01 ***p < 0.001). Abbreviations: QC-quinacrine, CTSL- cathepsin-L, LMP-Lysosomal Membrane Permeabilization, MOMP-Mitochondrial Outer Membrane Permeabilization, tBid- truncated Bcl-2 family member(Bid), p62- ubiquitinbinding protein 62, Cyt c- Cytochrome-c, ATG5- Autophagy Related 5, LC3B- Autophagy marker protein Light chain 3B, PARP- Poly (ADP-ribose) polymerase.

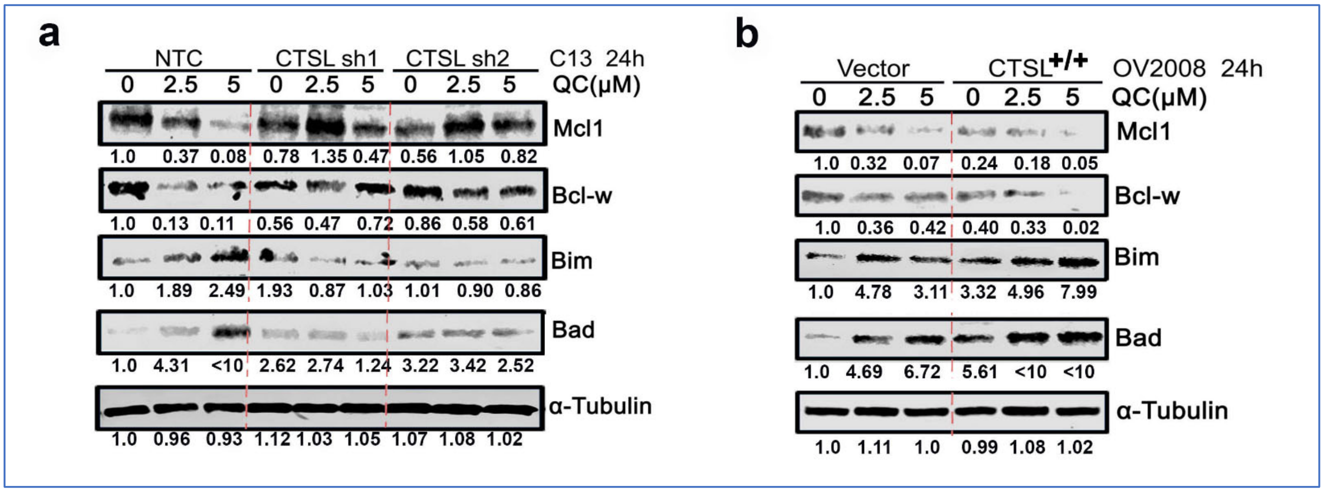

Figure S7: QC triggered CTSL disrupts pro and antiapoptotic homeostasis in ovarian cancer cells. Immunoblot analysis was performed in (**a**) CTSL KD sh1/sh2 and NTC control transfected C13 cells and (**b**) control vector and CTSL-overexpressed OV2008 in 24 hr QC treated cells using anti- Mcl1, Bcl-w, Bim and Bad. α-tubulin was used as loading control.
